# Scoring Performance on the Y-Balance Test Using a Deep Learning Approach

**DOI:** 10.3390/s21217110

**Published:** 2021-10-26

**Authors:** Manuel Gil-Martín, William Johnston, Rubén San-Segundo, Brian Caulfield

**Affiliations:** 1 Speech Technology Group, Information Processing and Telecommunications Center, E.T.S.I. Telecomunicación, Universidad Politécnica de Madrid, 28040 Madrid, Spain; ruben.sansegundo@upm.es; 2 Insight Centre for Data Analytics, University College Dublin, Belfield, Dublin 4, Ireland; william.johnston@insight-centre.org (W.J.); b.caulfield@ucd.ie (B.C.); 3 School of Public Health, Physiotherapy and Sports Science, University College Dublin, Belfield, Dublin 4, Ireland

**Keywords:** wearable sensors, Y Balance Test, time series data, recurrent neural networks

## Abstract

The Y Balance Test (YBT) is a dynamic balance assessment typically used in sports medicine. This work proposes a deep learning approach to automatically score this YBT by estimating the normalized reach distance (NRD) using a wearable sensor to register inertial signals during the movement. This paper evaluates several signal processing techniques to extract relevant information to feed the deep neural network. This evaluation was performed using a state-of-the-art human activity recognition system based on recurrent neural networks (RNNs). This deep neural network includes long short-term memory (LSTM) layers to learn features from time series by modeling temporal patterns and an additional fully connected layer to estimate the NRD (normalized by the leg length). All analyses were carried out using a dataset with YBT assessments from 407 subjects, including young and middle-aged volunteers and athletes from different sports. This dataset allowed developing a global and robust solution for scoring the YBT in a wide range of applications. The experimentation setup considered a 10-fold subject-wise cross-validation using training, validation, and testing subsets. The mean absolute percentage error (MAPE) obtained was 7.88 ± 0.20%. Moreover, this work proposes specific regression systems to estimate the NRD for each direction separately, obtaining an average MAPE of 7.33 ± 0.26%. This deep learning approach was compared to a previous work using dynamic time warping and k-NN algorithms, obtaining a relative MAPE reduction of 10%.

## 1. Introduction

Dynamic balance refers to the ability to maintain equilibrium while performing actions that include movements of the center of mass outside of the base of support. In particular, the Y Balance Test (YBT) is a test for assessing dynamic balance control, which has been widely used in clinical practice and research [1]. For example, YBT has been used for determining a person’s risk for injury [2] or return to sport readiness [3]. This test assesses performance during single-leg balance while reaching in three directions (anterior, posteromedial, and posterolateral). The traditional method for scoring the YBT is the normalized reach distance (NRD), which is obtained by measuring the distance an individual can reach in each of the three directions, normalized by leg length. Inertial measurement units (IMUs) are now being used to capture movement quality during the reaching tasks, providing a more sensitive approach to measuring dynamic balance performance. These IMUs provide a new opportunity to estimate the NRD directly from the sensor data and score the YBT, by developing a fully automated system. Although the NRD formula and setup are easy to assess, obtaining the NRD with the equipment requires the subjects to attend to the physiotherapist or sports center to perform the YBT. The equipment is cumbersome to move. Evaluating the YBT automatically using a back inertial sensor facilitates its evaluation, allowing subjects to supervise their evolution without the need of attending the sport center neither the help of the physiotherapist. The inertial signals could be used for feeding a deep learning architecture. The architecture output generates an estimation of the NRD, allowing to score the performance of the YBT.

Wearable inertial sensors have been widely used in sports science and medicine postural control applications. Postural control assessments have been frequently used for performance testing [4], injury risk screening, injury rehabilitation, and assessment of readiness to return to play [5]. Previous studies have been focused on evaluating the validity and reliability of assessment protocols in laboratory environments [6]. For example, a previous work [7] demonstrated that inertial sensor-derived 95% ellipsoid volume (95 EV) measure could capture alterations in dynamic balance control, which were not detected by traditional reach distances alone, and distinguish pre-fatigue and post-fatigue dynamic balance control for all three reach directions. In addition, another previous study [8] analyzed inter-session test-retest reliability of quantified YBT variables using a single lumbar inertial sensor, providing a reliable measure of balance performing across all three reach directions between tests performed in two different weeks. The authors analyzed the following YBT variables: NRD, 95 EV, ranges of pitch, roll, and yaw, and root mean square, sample-entropy, area under the curve of the fast Fourier transform and variance of the tri-axial gyroscope and accelerometer signals and magnitudes of the lumbar sensor. Regarding injuries related to sports, another previous work [2] demonstrated that poor dynamic balance performance, measured by a lumbar inertial sensor during the YBT, was significantly associated with a subsequent concussion injury. The authors used data from an elite rugby union, and they concluded that individuals with poorer balance performance were three times more likely to sustain a sports-related concussion.

Regarding classification tasks related to dynamic balance, a previous work [1] discriminated between the three YBT reach directions and between pre and post-fatigue balance performed during the YBT. The authors recorded data from fifteen subjects performing YBT on the dominant leg at 0, 10, and 20 min. They used features extracted from a lumbar sensor and a random forest classifier. They obtained 97.80% of accuracy, 97.86 ± 0.89% of sensitivity, and 98.90 ± 0.56% of specificity for the reach direction classification task. Regarding fatigue, “normal” and “abnormal” balance performances were classified with an accuracy of 61.90–71.43%, sensitivity of 61.90–69.04%, and specificity of 61.90–78.57% depending on which reach direction was chosen.

Regarding regression tasks related to dynamic balance, a previous study in the literature [3] proposed and evaluated a machine learning approach based on the k-nearest neighbour (k-NN) algorithm and dynamic time warping method to estimate the NRD over a dataset with 29 young healthy adults [9]. This study used data from 21 subjects for training and validating the model and data from the remaining eight subjects for testing it. The authors observed that the *Z*-axis from the lumbar accelerometer was the most informative signal. This previous work used a 10-fold cross-validation for the training and validation procedure and evaluated the final model over the eight unseen subjects. The results reported a mean absolute percentage error (MAPE) of 6.24% and 8.02% over the training and testing subsets, respectively. Another recent work [10] studied kinematic and kinetic predictors of YBT performance for each direction using data from 31 healthy subjects. The authors built a stepwise regression model with specific variables such as flexion or rotation of the knee, hip, ankle, or torso. Knee flexion and torso contralateral rotation explained 45.8% of the variance in anterior reach direction, the combination of hip flexion, ankle dorsiflexion, and external rotation explained 76.9% of the variance in posteromedial reach direction, and hip flexion and pelvis contralateral rotation explained 69.6% of the variance in the posterolateral reach direction. The conclusions of this work remarked that hip and knee joint moments in the sagittal and frontal planes were critical for YBT performance.

Deep learning algorithms have been widely integrated into human activity recognition (HAR) systems [11] and have outperformed traditional machine learning techniques. Some of these deep learning architectures are composed of convolutional layers which could capture spatial and temporal dependencies of inputs through convolutions with filters. Other architectures are composed of recurrent layers which could learn the evolution of a sequence through internal memory cells. Within HAR systems, gesture recognition has been achieved using these architectures or a combination of both [12,13]. This way, motion during a YBT could be considered as a gesture, so deep learning approaches could generate a robust model based on the performed movement during the YBT excursions. To the best of the authors’ knowledge, in the literature, there is not a previous study using deep learning algorithms (recurrent neural networks) for estimating YBT distances. This would be the most important contribution of this paper.

This paper addresses the challenge of automatically estimating the NRD of the YBT. The main contributions of the paper are the following:

- Analysis of the YBT NRD estimation task using data from a wide variety of subjects.
- Proposal and evaluation of a deep learning approach to estimate the NRD of the YBT by modeling the temporal pattern of the movement. In this analysis, several normalizations and input formats were analyzed. This approach automatically evaluates the YBT using a back inertial sensor, allowing subjects to supervise their evolution without the need of attending the sport center neither the help of the physiotherapist.
- Comparison of two approaches for NRD estimation: creating a unique robust model for all directions or building specific systems to estimate the NRD for each direction.
- Description of a detailed analysis of correlations between real and estimated NRD.

This study was performed over a dataset with YBT from 407 different subjects using a subject-wise cross-validation strategy. To the best of the authors’ knowledge, this dataset is the biggest in the literature. Moreover, a subset of this dataset was used for comparison with previous results.

## 2. Materials and Methods

This section describes the YBT, the dataset used for the experiments, the signal processing techniques, and the deep learning approach based on LSTMs.

### 2.1. Y Balance Test and Collection Protocol

The YBT is an instrumented substitute of the Star Excursion Balance Test (SEBT), efficient for measuring dynamic postural control. The YBT is a clinical assessment that is traditionally scored by measuring the reach distance [1] (NRD). This distance provides an objective measurement.

**Figure 1:**
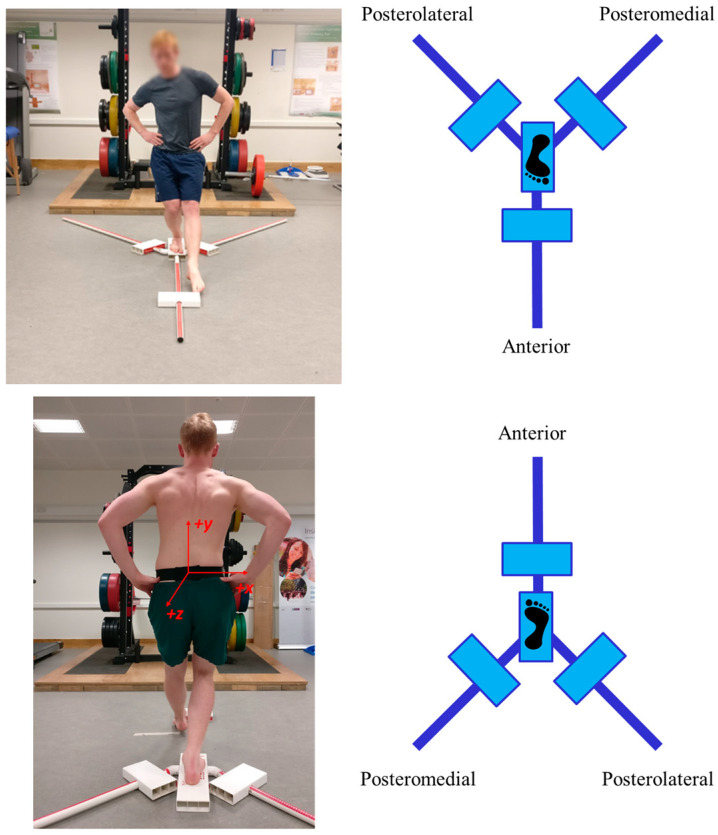
A YBT excursion in operation, a diagram of the directions [3], and the lumbar inertial sensor orientation and location.

The YBT consists in switching from an initial bilateral to unilateral stance and maintaining controlled balance while using one leg to perform a maximal reach excursion with the non-stance limb in the three standardized directions [14]. Figure 1 shows an individual performing a YBT excursion, a diagram of the anterior, posteromedial, and posterolateral directions and the location and orientation of the lumbar sensor. The subject must return to the starting bilateral stance in a controlled way. A trial is considered a failure if any of these situations occurs: the subject removes his hands from the hips, contacts the ground, uses the block for support, raises the stance leg heel, or kicks the slider forward for extra distance.

### 2.2. Dataset

The dataset contains YBT recordings from 407 subjects (aged 23.1 ± 6.6 years; height 179.8 ± 42.1 cm; weight 89.3 ± 21.1 kg; left leg length 96.6 ± 7.6 cm; right length 96.9 ± 6.4 cm). The dataset contains data from 407 subjects from different cohorts: 107 professional Rugby Union athletes, 32 Intercounty Gaelic Football athletes, 104 young healthy adults (18–40 years), 18 healthy middle-aged adults (40–64 years), 97 NCAA Division 1 American football, and 49 NCAA Division 1 ice hockey players. All participants were healthy subjects and they self-reported no musculoskeletal or neurological impairments at the time of testing. In this sense, the dataset offers data from a wide variety of population and some athletes from different sports. Data were collected in a standardized manner which has previously been detailed in the literature ([2,7,8,15]). The university research ethics board granted ethical approval, and all subjects gave informed consent before the completion of the testing protocol.

The participants were informed about the YBT, and they performed several practice trials before the data collection. Each session consisted of three YBT excursions in three different directions (anterior, posteromedial, and posterolateral) and with the two legs in a randomized order. From each subject, 18 recordings were obtained per session: 3 YBT excursions × 3 directions × 2 stance legs. However, some of the YBT samples were missing in the dataset. The YBT reach distance and sensor data were collected for each excursion. Data were labelled by measuring the reach distance over the experimental platform. First, the researchers measured the leg length as the distance from the anterior-superior iliac spine to the most distal aspect of the medial malleolus [15]. Second, once the subject reached the distance by moving the slider over the platform, they write down the distance and normalized it using the subject’s leg length using Equation (1).

(1) $$\mathrm{Normalized}\;\mathrm{Reach}\;\mathrm{Distance}=\frac{\mathrm{Raw}\;\mathrm{Reach}\;\mathrm{Distance}\;\left(\mathrm{cm}\right)}{\mathrm{Leg}\;\mathrm{Length}\;\left(\mathrm{cm}\right)}*100$$

The total number of samples included in this dataset is 7262 (2427 from the anterior direction, 2411 from the posteromedial direction and 2424 from the posterolateral direction), corresponding to 407 subjects.

**Figure 2:**
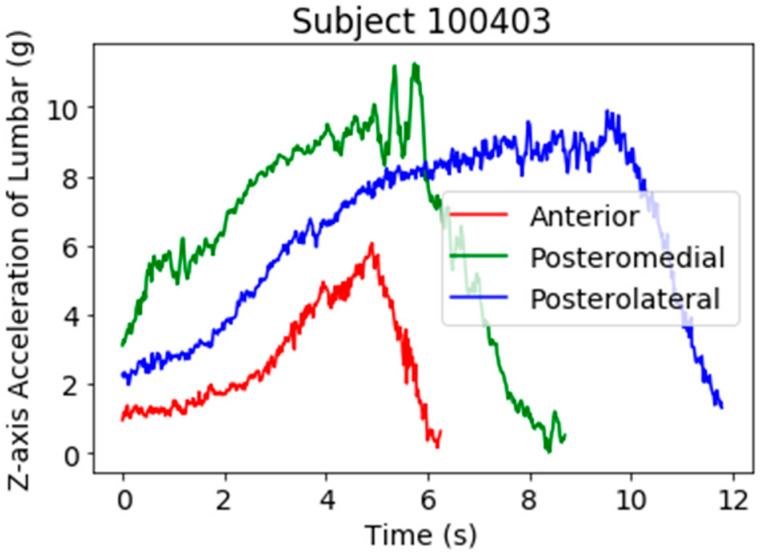
*Z*-axis lumbar acceleration signal of YBT excursions from subject 100403.

These subjects were wearing a single inertial sensor (Shimmer3, Dublin, Ireland), which provided an accelerometer, gyroscope, and magnetometer in three dimensions. The sensor was mounted at the level of the fourth lumbar vertebra, in line with the top of the iliac crests and secured using a custom-made elastic belt to closely match the acceleration of the body’s center of mass during the YBT excursions. Figure 1 shows a subject wearing the belt where the lumbar sensor was attached, and the *Z*-axis pointed backwards. The inertial sensor was connected via Bluetooth to an Android tablet (Galaxy Tab 2, Samsung, Seoul, Korea) operating a custom-made application and configured to collect tri-axial accelerometer (±2 g), tri-axial gyroscope (±500 °/s) and tri-axial magnetometer (±1 gauss) data at a sampling frequency of 51.2 Hz during each YBT reach excursion. The Shimmer3 sensor was calibrated prior to data collection following the standardized procedure outlined by the manufacturer [16]. These data acquisition parameters were defined based on pilot testing and previous work investigating the utility of inertial sensors in the evaluation of exercise technique and balance [1,7,17]. Shimmer sensor is a general-purpose device but could be used for clinical postural balance purposes since it provides accurate measurements of inertial signals. In this work, we used the *Z*-axis from the lumbar accelerometer as suggested by [3]. This previous work concluded that this signal was the most informative one and no improvement was obtained when including additional signals. We observed that *Z*-axis acceleration signal have a greater standard deviation during the YBT (mean 5.07, std 2.85 g) compared to X (mean −0.09, std 1.64 g) and Y (mean 7.57, std 2.17 g) axes. A higher variability can provide more information about the movements and about the NRD. This aspect has been verified evaluating the system with other signals. Figure 2 shows Z acceleration of YBT excursions from subject 100403 in the three directions (anterior in red, posteromedial in green, and posterolateral in blue). In this figure, it is possible to observe a common pattern of the YBT excursions: a gradual increase of acceleration and final braking. In addition, the figure shows that the acceleration signal reaches higher values for directions posteromedial and posterolateral compared to the anterior direction.

**Figure 3:**
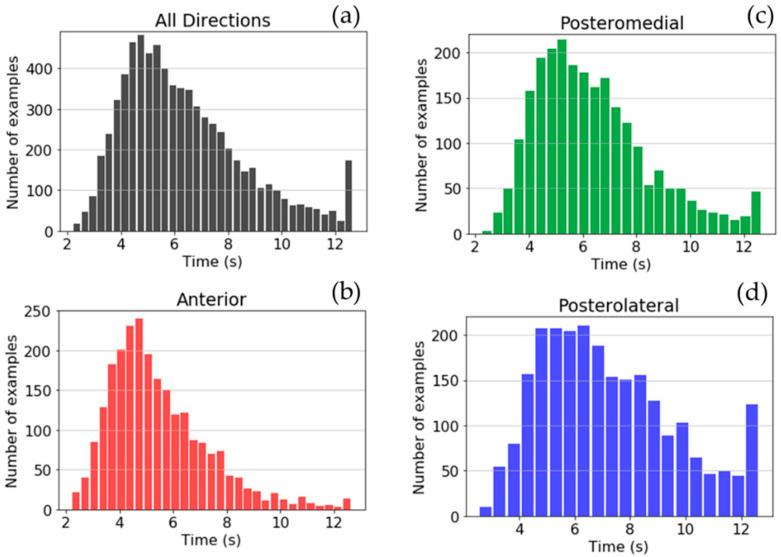
Histograms of YBT excursions depending on the duration for all directions: (**a**), anterior direction (**b**), posteromedial direction (**c**), and posterolateral direction (**d**).

These excursions have different lengths: from 113 to 648 data-points (2.2 s to 12.7 s), with a mean of 324.83 data-points (6.3 s) and a standard deviation of 115.84 data-points (2.3 s). Figure 3 shows the histograms of YBT excursions of the dataset depending on duration. The specific histograms for each direction show that YBT excursions of the anterior direction usually last less time than posteromedial and posterolateral excursions. These histograms also show that YBT excursions in all directions could last until approximately 12 s.

**Figure 4:**
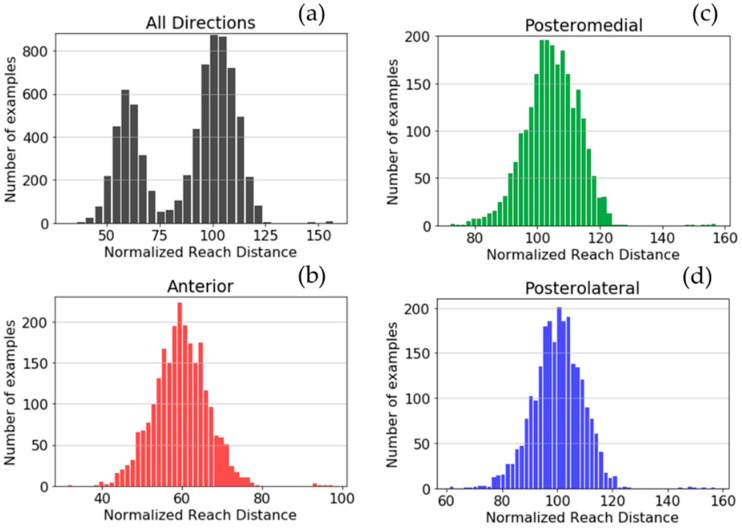
Histograms of YBT excursions depending on the NRD for all directions (**a**), anterior direction (**b**), posteromedial direction (**c**), and posterolateral direction (**d**).

Figure 4 shows the histograms of YBT excursions of the dataset depending on the NRD. The histogram in the YBT excursions of all directions shows values between 40 and 157, and it suggests that there exist two Gaussian profiles.

The specific histograms for each direction show that YBT excursions of each direction have a different mean and standard deviation of the reach distance: 59.65 ± 6.73, 104.63 ± 8.66, and 100.27 ± 9.10 for anterior, posteromedial, and posterolateral, respectively.

### 2.3. Signal Processing and Deep Learning Approach

**Figure 5:**
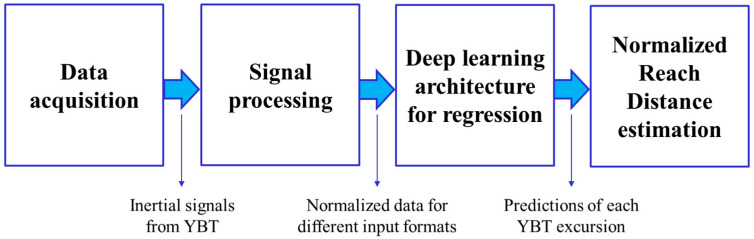
HAR framework of this work including the outputs of the intermediate modules.

HAR systems typically use a general-purpose framework [17] with several modules for activity recognition tasks, which could be extended for regression tasks. This framework contains two main modules: a signal processing module that extracts the features or transforms the signals and a machine or deep learning system that models and could estimate a specific measure for each sample. This general-purpose framework of HAR systems could be adapted to our regression system. Figure 5 presents the sequence of modules for the HAR framework used in this work, mentioning the outputs of the intermediate modules.

The raw signals were normalized considering several possibilities. The *Z*-axis lumbar acceleration signal was normalized by the mean along with all samples in each example (by examples), all samples of the excursions in a specific direction (direction), or all samples of all excursions from the same subject (subject), respectively

As mentioned, the length of the time series is different for each YBT excursion while the inputs to the deep learning architecture have a fixed size. Because of this, after normalizing the signal, zero padding was used at the beginning of the signal to have examples of the same duration: 650 points, which correspond to 12.7 s. These initial zeros did not affect the system performance because recurrent neural networks could learn this type of patterns, obtaining information about the YBT duration.

After zero-padding, we extracted features. In this process, we evaluated two possibilities: using raw recordings directly (leaving to the deep learning architecture the process of learning features automatically), and handcrafted features from YBT sub-windows.

**Figure 6:**
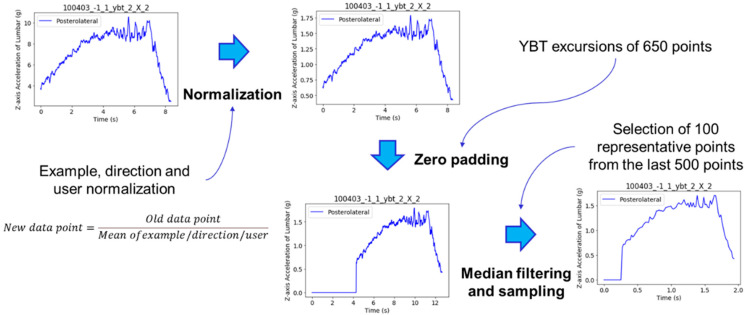
Complete signal processing for the YBT excursions.

For the raw data approach, to reduce the number of inputs to the deep learning architecture, we selected 100 representative points from the last 500 points of each example after downsampling (filtering and sample selection). This way, we obtained YBT examples of 100 data points. We followed this technique because a sequence of 650 points is too long to be analyzed by a recurrent layer. Figure 6 shows the complete signal processing for the YBT excursions, showing the process of an example of posterolateral direction from subject 100403.

**Figure 7:**
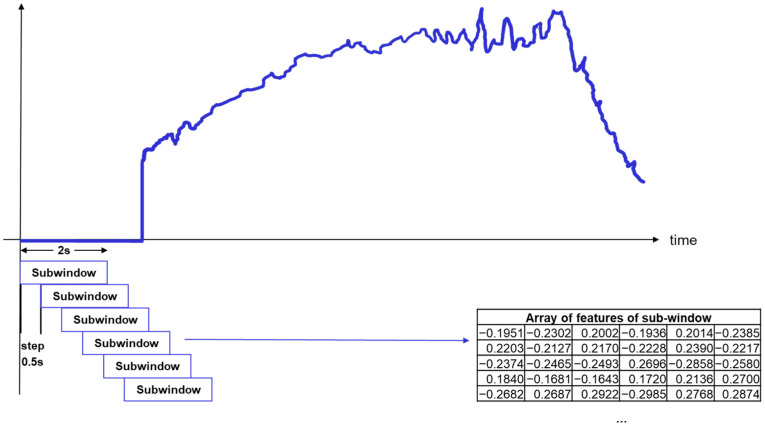
YBT windowing and features computation of sub-windows.

Regarding the feature extraction process, each YBT example was subdivided into 2 s subwindows with a step of 0.5 s (overlap of 1.5 s) after the normalization and zero padding processes. For all 12.7 s excursions, 22 sub-windows of 186 features were obtained. Afterward, we extracted handcrafted features for each sub-window to learn a temporal model from the evolution of the features through the YBT excursion. Figure 7 shows the sub-windowing process for a YBT example and the extraction of features of the subwindows.

For features computation, we used a time series feature extraction library [18] to compute over 60 different features extracted across temporal, statistical, and spectral domains for each of the sub-windows. Barandas et al. [18] explained the computation details for each feature. The features are grouped into three sets, as shown in Table 1: temporal domain, statistics, and spectral domain.

Thanks to the Fourier analysis, it is possible to detect fast signals variations that are related to vibrations produced by the exercise stress. These oscillations increase the energy at high frequencies. In addition, slow movements, which could be related to the beginning and the end of the exercise, are associated with energy in low frequencies. For these reasons, we included features from the fast Fourier transform in the spectral domain subset (Table 1).

**Figure 8:**
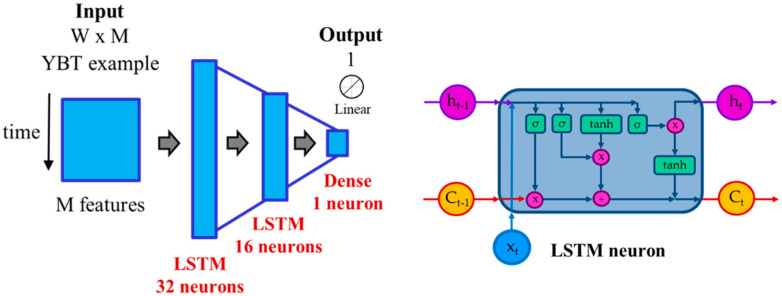
RNN architecture and structure inside an LSTM neuron.

The deep learning architecture was composed of a time modeling subnet and an additional fully connected layer for estimating the normalized reach distance (regression). The first subnet modeled the time patterns using recurrent layers while the second part of the network estimated the NRD. The output of the architecture was the estimated NRD for every YBT excursion. We used the mean squared error (MSE) as loss metric and the Adagrad as the optimizer, with parameter-specific learning rates that were adapted relative to how frequently a parameter gets updated during training. The deep learning architecture had two long short-term memory (LSTM) layers with 32 and 16 neurons, respectively, and a final dense (fully connected) layer with one neuron and a linear activation function. The architecture included intermediate dropout layers (20%) after recurrent layers to avoid overfitting during training. These recurrent layers are capable of learning long-term dependencies in sequence prediction problems, extracting the temporal model of each example, and generating a model based on the pattern of the YBT excursion. Figure 8 represents the RNN architecture based on LSTMs, which was optimized by evaluating the system performance over validation subsets and the structure inside an LSTM neuron. This architecture used a 2 D input considering W sub-windows × M features as dimensions. In the case of using raw signals directly, the dimensions were W excursion length × M = 1 (Z acceleration values). This figure also shows that the LSTM neuron is composed of sigmoid (σ) and hyperbolic tangent (tanh) layers and how it manages the internal memory. x_t_, h_t_, and C_t_ denote respectively the input, the output, and the cell state memory of the module, and h_t-1_ and C_t-1_ denote the output and the cell state memory of the previous module. This architecture has been implemented in Python using Keras with Tensorflow as backend. In the experiments, other tools like sklearn have been used. Table 2 details the different layers of the architecture. The deep learning architecture was separately optimized regarding the different input formats: raw data and features. The final architecture was the same in both cases, which was optimized using a validation subset: the best performance over the validation subset was obtained using a learning rate of 0.02, a batch size of 100, and 50 epochs.

## 3. Results

This section describes the evaluation metrics used in this work and discusses the results obtained in the experiments performed in these analyses.

### 3.1. Evaluation Metrics and Validation

As commented in the introduction, this paper reports the results of estimating the NRD in YBTs. For this regression task, we computed the mean absolute percentage error (MAPE) according to Equation (2), where $y_{i}$ is the real NRD, ${\hat{y}}_{i}$ is the predicted NRD, and N is the number of samples. This metric was used to compare the real NRD and the estimated one, and the results are presented with confidence intervals obtained at a confidence level of 95% (Equation (3)). In this equation, N is the number of samples, *Error* a specific error, and its standard deviation σ. If there is no overlap between the confidence intervals of different approaches, this fact suggests that the results are significantly different:

(2) $$\mathrm{MAPE}=\frac{\sum_{i=1}^{N}\frac{\left|y_{i}-{\hat{y}}_{i}\right|}{y_{i}}}{N}$$

(3) $$Error\;\left(95\%\right)=Error\pm 1.96*\frac{\sigma }{\sqrt{N}}$$

In addition, this work presents correlation coefficient measures between the real and the predicted NRDs. Equation (4). defines the Pearson correlation, where the covariance of *X* and *Y* is divided by the product of the standard deviation of *X* and *Y*. *X* and *Y* are the real and estimated NRD, respectively:

(4) $$\rho_{X,Y}=\frac{cov\left(X,Y\right)}{\sigma_{X}\sigma_{Y}}$$

**Figure 9:**
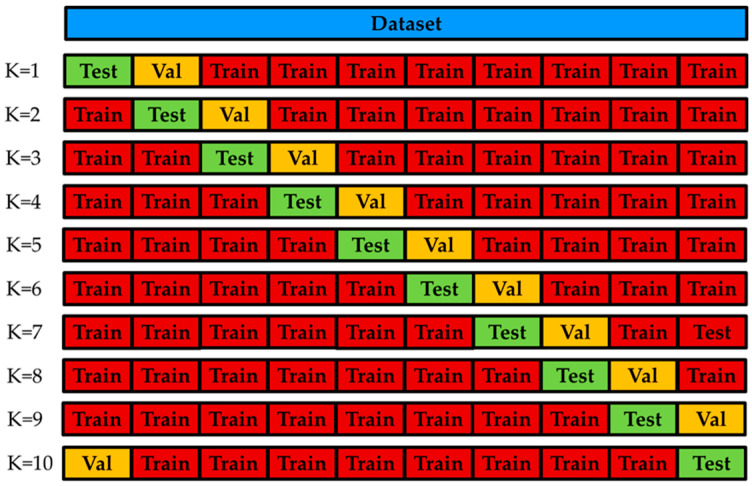
Round-robin strategy for 10-fold Subject-Wise cross-validation.

Regarding the cross-validation strategies used in the experiments, we performed a 10-fold subject-wise cross-validation strategy: data are divided in such a way that the data from the same subject are contained only in one subset (training, validation, or testing). In each iteration data from subjects were arranged in 10 folds, eight folds were used to train the system, one fold to optimize the system and the remaining fold to test the system. This process was repeated 10 times modifying the training, validation and testing subsets by a round-robin strategy as shown in Figure 9.

The results presented in this work were average values obtained throughout the cross-validation procedure. A validation subset was used for adjusting the main parameters of the deep learning system, and then this validation subset was included in the training subset to train the final model before the evaluation over the testing subset.

### 3.2. Experiments

This section includes the experiments and analyses performed over the YBT dataset. In these experiments, we analyzed the impact of the data normalization and input format of the YBT excursion over the regression performance. Afterward, we compared two procedures to estimate the NRD in the YBT excursion: using a unique system for all directions or using specific systems for each direction.

#### 3.2.1. Data Normalization and Input Format

This subsection shows the regression performance depending on the data normalization applied to the inertial signals. Table 3 illustrates the validation and testing MAPE results when no input normalization was applied or when different normalizations were computed over the raw data excursion: example, direction or user normalization. As shown, selecting the appropriate data normalization is crucial for estimating the NRD of YBT because not all normalizations work in the same way for this task. For example, performing an input normalization using the data for each example or direction could be counterproductive. This table suggests that each subject could have specific energy when performing the YBT in all directions that could be different from the one used by another one. In this sense, normalizing the data of each subject separately improved the performance of the NRD estimation. We obtained a significant difference when performing a user normalization of data, so, from this point, the rest of the experiments were done using a user normalization.

We also analyzed the duration of the raw YBT excursions from 50 to 500 data points, and we observed that the error performance kept stable, as shown in Table 4. This way, we used 100 points as compromise between time resolution and sequence complexity to be learnt by the LSTM layers.

As mentioned in the dataset description section, the *Z*-axis acceleration signal was showed the greatest standard deviation during the YBT, providing more variability about the movements. To verify the contribution of the other axes, we added *X*-axis and *Y*-axis accelerations, but we observed a worse performance: the system provided a validation MAPE of 9.22 ± 0.08% and a testing MAPE of 9.06 ± 0.26%, compared to 8.91 ± 0.08% and 8.78 ± 0.22% when using only the *Z*-axis acceleration. *X*-axis and *Y*-axis accelerations do not contribute to improve the system performance and can be discarded to have a simpler system.

Table 5 illustrates the validation and testing MAPE results depending on the input format using a user normalization: raw, or features. For the case of using handcrafted features at sub-windows, we evaluated different sub-window lengths: 1 s, 2 s and 3 s. The first conclusion is that we obtained a lower prediction error when using handcrafted features as inputs to the deep learning architecture. Regarding the sub-windows length, the lowest validation MAPE was obtained when using handcrafted features from 2-s sub-windows. The deep learning architecture was able to learn a better model of the YBT excursions to estimate the reach distance when it was fed by specific features instead of the raw data directly. From this point, the rest of the experiments were done using handcrafted features obtained from 2 s subwindows.

Table 6 includes the validation and testing MAPE results depending on the subset of features used. Results suggested that the temporal-domain features were the most informative. When using only these features, the performance was similar to the case of using the whole set. In this sense, if an optimized system in terms of resources consumption is required, the suggestion would be to use only temporal-domain features.

**Figure 10:**
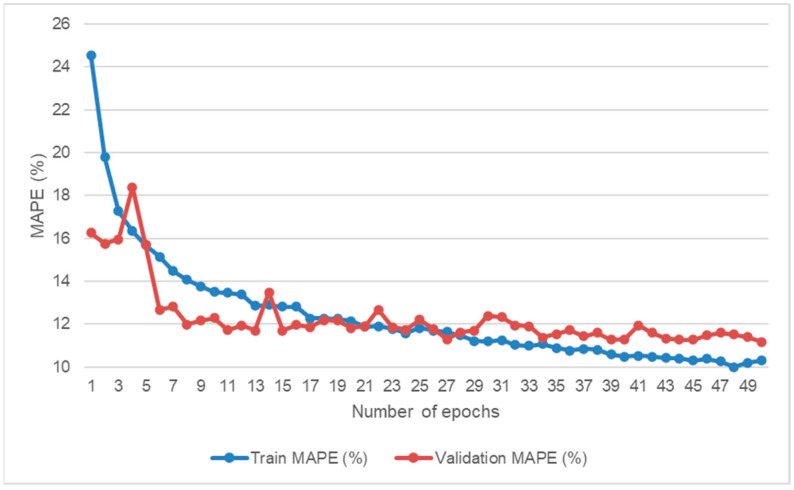
Training and validation MAPE during the RNN training.

As an example of error evolution through the training process, Figure 10 shows the training and validation MAPE through the 50 epochs of one of the validation folds inside the 10-fold subject-wise cross-validation strategy when all features were used.

This figure shows how the training error is continuously decreasing as increasing the number of epochs while the validation error oscillated until becoming a stable value around 50 epochs.

#### 3.2.2. Unique System for All Directions vs. Specific Systems for Each Direction

In the next analysis, we evaluated two procedures for estimating the NRD: by using a unique system for all the directions or considering specific systems for each direction. The system characteristics are those that obtained the best performance in previous sections.

A unique system was trained with the YBT excursions of all the directions, which was able to indirectly detect the direction of the excursion and estimate the reach distance for each example. In this case, the variability of the reach distance values was high.

Specific systems for each direction were focused on estimating the reach distance using the examples of a single direction. The target is to train a specific model for each direction. This approach solved more specific tasks where the variability of the output is lower.

**Figure 11:**
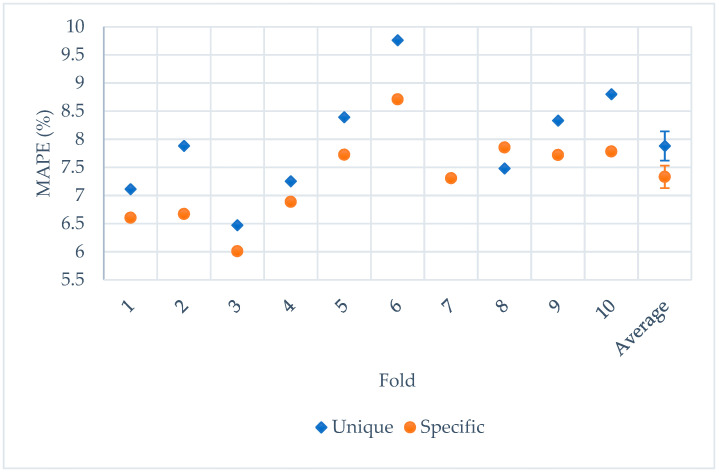
Results from a unique system and specific systems.

Figure 11 shows the testing MAPE results per fold and the average results for both procedures. Results suggest that a specific system for each direction could surpass the performance of a unique system for all directions, obtaining a significant difference between the average results (7.88 ± 0.20% for the unique system and 7.33 ± 0.26% for the specific systems). When using a unique system, this system was trained with higher variability of examples: different acceleration ranges and NRDs. When building specific systems trained with data from the same direction, the variability is lower, and the regression problem is less difficult.

Table 7 indicates the testing MAPE obtained for each direction depending on the approach. We could observe that the error performance of each direction could be reduced, being significant for the anterior direction.

An additional experiment was performed to compare the proposed method to the previous study [3] mentioned in the related work section. This previous work estimated the NRD using a k-NN algorithm and dynamic time warping method over a dataset with 29 young healthy adults [9]. The deep learning system was evaluated using the same experimental setup: 21 subjects for training and validating the model and eight subjects for testing it. The previous work achieved a test MAPE of 8.02%, while the proposed methods obtained a test MAPE of 7.59 ± 1.51% and 7.27 ± 1.75% using a unique system for all directions and a specific system for each direction, respectively. Results suggest that the deep learning approach could reduce the regression performance. In this case, there is not a significant difference between the results because the number of testing samples was only 143 YBT excursions. Previous work was based on measuring the dynamic time warping between sequences for estimating the reach distance. However, the LSTM layers (proposed in this work) obtained better time models based on the evolution of these sequences.

#### 3.2.3. Results Analysis

**Figure 12:**
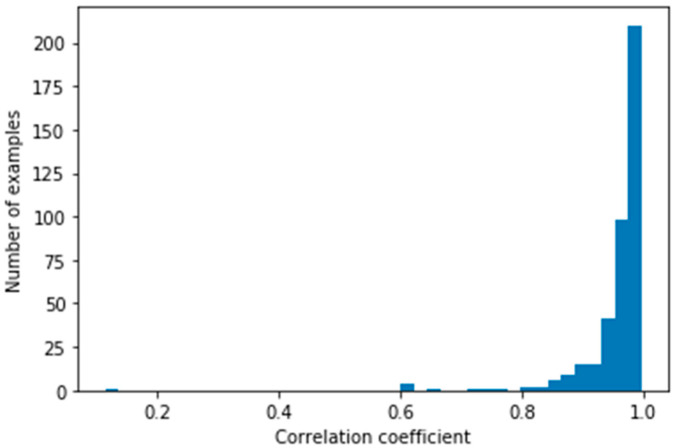
Histogram of correlation per subject.

If we analyzed the MAPE and the correlation between the real and the predicted NRD for each subject, we could focus on the most challenging subjects and discuss the reasons of this poor performance. Figure 12 shows a histogram of correlation per subject, where it is possible to observe that most of the subjects reached a high correlation. This distribution has a mean of 0.956 and a standard deviation of 0.068. Although most of the subjects (369) obtained a correlation higher than 0.9, some isolated subjects (nine) obtained a correlation lower than 0.8.

**Figure 13:**
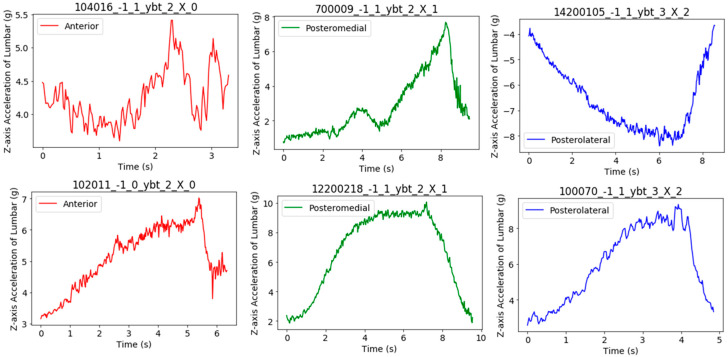
YBT excursions from subjects with the lowest correlation (**top**) and subjects with the highest correlation (**bottom**).

Figure 13 shows YBT excursions from subjects with the lowest correlation on the top and subjects with the highest correlation on the bottom. It is possible to observe that the excursions on the top do not follow the general pattern described in Figure 2: they presented imbalances, a sharp increase of acceleration, or an opposite pattern of the movement. Some subjects reached the distance after several attempts while moving around the measurement platform, which could lead to imbalances or a sharp increase of acceleration. However, the opposite pattern of movement was caused by a turned sensor while data collection.

In contrast, the YBT examples from the subjects with the highest correlation displayed an appropriate movement pattern of the YBT for the three directions, which was observed when the dataset was presented. This reveals the evidence about how important is carefully supervising the data collection protocol of YBT because excursions with anomalous movement patterns could disturb the model generation and affect the NRD of those examples.

**Figure 14:**
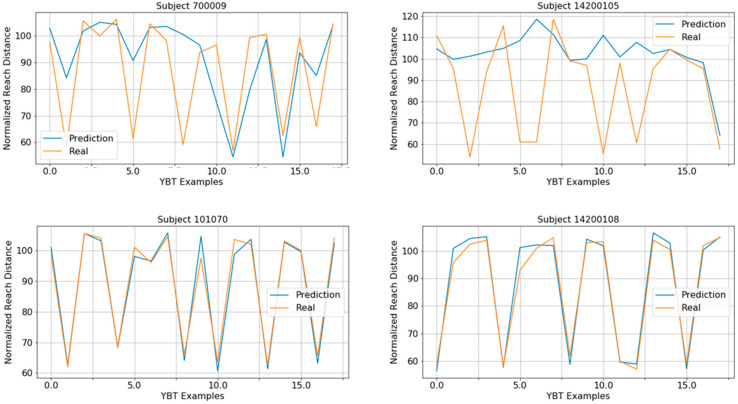
Real and predicted NRD of YBT from subjects with high MAPE (**top**) and subjects with low MAPE (**bottom**).

Figure 14 shows the real and predicted NRD of YBT from subjects with high MAPE and low MAPE. It is important to highlight that for subjects with anomalous movements (Figure 13) the NRD prediction does not fit the ground truth. This is noticeable in subjects with IDs 700009 and 14200105 (top of Figure 14), where the regression task failed. However, there are other subjects with a highly accurate estimation, as shown at the bottom of Figure 14.

## 4. Discussion

The results obtained in the previous section validate the use of deep learning techniques to score the YBT by estimating the NRD. This approach has been evaluated through a dataset that contains recordings from a wide range of subjects (407 subjects aged from 18 to 64 years). This set of different people could be used to create a robust model that could generalize to other subjects. However, a user normalization of recordings is required to build this general model since each person has distinct energy while performing the YBT excursions.

Regarding the input format, this manuscript evaluated raw and handcrafted features as inputs to the deep learning architecture. Results suggested that the recurrent layers architecture could boost the NRD estimation performance when it was fed with specific features from 2-s sub-windows of the YBT excursions instead of leaving the network directly learns from raw recordings. This aspect could happen in complex tasks, where extracting features for representing the recordings is worthy. We also observed that the temporal-domain features were the most informative ones, reaching a similar performance than using the entire set of features.

Training specific systems with data from the same direction allows a better modeling of the regression problem because this training use data with lower variability. These specific systems reach significantly better performance compared to training a unique model for all directions. The supervision of subjects during the YBT collection protocol is crucial to avoid anomalous recordings that could disturb the modeling process. These recordings could hinder the training and they would obtain a worse NRD estimation error.

## 5. Conclusions

Dynamic balance assessments are crucial in clinical practice and research regarding risk screening and injury rehabilitation. This work analyses the YBT NRD estimation task using a dataset of 407 different subjects and a 10-fold subject-wise cross-validation strategy. This wide variety of healthy subjects includes data from young and middle-aged volunteers and athletes from different sports, which allows developing a global and robust solution for scoring the YBT in a wide range of possible scenarios. We analyzed different normalizations and input formats to automatically score the YBT by estimating the NRD. We observed that the combination of a user normalization of data and feature computation of sub-windows from each YBT example could boost the estimation performance using a deep learning approach. This deep learning architecture is composed of LSTM layers which were able to extract a temporal model of each example through the evolution of the YBT sub-window features. This approach obtained a testing MAPE of 7.88 ± 0.20%. In addition, building a specific system to estimate the NRD for each direction could reduce this performance, reaching 7.33 ± 0.26% of MAPE. This work also highlighted that it is important to carefully supervise the data collection protocol of YBT because the subjects with the lowest correlation between the real and predicted distances presented some imbalance, a sharp increase in acceleration, or opposite patterns of movement to what was typically observed. This deep learning approach achieves a relative MAPE reduction of 10% compared to previous work which used a machine learning procedure using the same experimental setup [3].

As future work, it would be interesting to create a system that automatically detects abnormal YBT excursions right after the collection protocol. This way, it would be possible to repeat during the collection protocol the YBT excursions that do not fit the mentioned appropriate movement pattern that allows better estimating the NRD. In addition, it would be interesting to develop specific systems for every cohort of subjects.

## Figures and Tables

**Table 1:** Features from different domains.

Temporal Domain	2nd Order Statistics	Spectral Domain
- Autocorrelation - Centroid - Mean absolute differences - Mean differences - Median absolute differences - Median differences - Distance - The sum of absolute differences - Total energy - Entropy - Peak to peak distance - Area under the curve - Absolute energy - Maximum peaks - Minimum peaks - Positive turning points - Neighborhood peaks - Negative turning points - Slope - Zero-crossing rate	- Histograms - Interquartile range - Mean absolute deviation - Median absolute deviation - Root mean square - Standard deviation - Variance - Empirical Cumulative Distribution Function (ECDF) percentile counts - Kurtosis - Skewness - Maximum - Minimum - Mean - Median - ECDF coefficients - ECDF percentiles	- FFT mean coefficients - Wavelet absolute means - Wavelet standard deviations - Wavelet variances - Spectral distance - Fundamental frequency - Maximum frequency - Median frequency - Spectral positive turning points - Max power spectrum - Spectral centroid - Spectral decrease - Spectral kurtosis - Spectral skewness - Spectral spread - Spectral slope - Spectral variation - Spectral roll-off - Spectral roll-on - Human range energy - Mel-Frequency Cepstrum Coefficients - Linear Prediction Coefficients - power bandwidth - Spectral entropy - Wavelet entropy - Wavelet energies

**Table 2:** Configuration details and number of parameters for all layers of the deep learning architecture using features from 2-s wub-windows as input.

Layer	Output Shape	Param #	Activation Function	Other Characteristics
Input	(-, 22, 186)	-	-	-
LSTM	(-, 22, 32)	28,032	-	kernel_initializer = “glorot_uniform”, return_sequences = True
LSTM	(-, 16)	3126	-	kernel_initializer = “glorot_uniform”, return_sequences = False
Dense	(-, 1)	17	Linear	kernel_initializer = “glorot_uniform”,
Output	(-, 1)	-	-	-

**Table 3:** Results of regression task using different normalization of raw data.

Input Normalization	Val MAPE (%)	Test MAPE (%)
None	9.55 ± 0.09	9.33 ± 0.25
Example	17.21 ± 0.15	17.21 ± 0.43
Direction	13.08 ± 0.11	12.12 ± 0.31
User	8.91 ± 0.08	8.78 ± 0.22

**Table 4:** Test MAPE using subject normalization over raw data depending on the number of data points of the YBT excursions.

Data Points	Test MAPE (%)
50	8.87 ± 0.23
100	8.78 ± 0.22
167	8.82 ± 0.23
250	8.87 ± 0.24
500	8.82 ± 0.24

**Table 5:** Results of regression task using different input format and user normalization.

Input Format	Val MAPE (%)	Test MAPE (%)
Raw	8.91 ± 0.08	8.78 ± 0.22
Features from 1 s subwindows	8.38 ± 0.07	8.00 ± 0.20
Features from 2 s subwindows	8.35 ± 0.07	7.88 ± 0.20
Features from 3 s subwindows	8.37 ± 0.07	8.08 ± 0.20

**Table 6:** Results of regression task using different subsets of features from 2-s sub-windows.

Subset of Features	Val MAPE (%)	Test MAPE (%)
All	8.35 ± 0.07	7.88 ± 0.20
Temporal domain	8.16 ± 0.07	7.87 ± 0.19
Statistical domain	8.69 ± 0.07	8.32 ± 0.20
Spectral domain	8.43 ± 0.07	8.12 ± 0.20

**Table 7:** Results for both strategies: unique system and specific systems for each direction.

Test MAPE (%) per Direction		
Direction	System	
	Unique model	Specific models
Anterior	10.27 ± 0.46	8.69 ± 0.29
Posteromedial	6.31 ± 0.22	6.28 ± 0.21
Posterolateral	7.04 ± 0.26	7.02 ± 0.25

## Data Availability

Y BALANCE TEST (YBT) DATASET. http://mlg.ucd.ie/ybt/; accessed date: 16 June 2021.
